# FTIR Spectroscopy Detects Intermolecular β-Sheet Formation Above the High Temperature T_m_ for Two Monoclonal Antibodies

**DOI:** 10.1007/s10930-020-09907-y

**Published:** 2020-07-12

**Authors:** Garrett Baird, Chris Farrell, Jason Cheung, Andrew Semple, Jeffery Blue, Patrick L. Ahl

**Affiliations:** 1 Merck & Co., Inc., MMD, West Point, PA USA; 2 Merck & Co., Inc., MRL, West Point, PA USA; 3 Merck & Co., Inc., MRL, Kenilworth, NJ USA

**Keywords:** Monoclonal antibodies, FTIR, Aggregation, Unfolding, Protein stability

## Abstract

The temperature-dependent secondary structure of two monoclonal IgG antibodies, anti-IGF1R and anti-TSLP, were examined by transmission mode Fourier Transform Infrared (FTIR) spectroscopy. Anti-IGF1R and anti-TSLP are IgG monoclonal antibodies (mAbs) directed against human Insulin-like Growth Factor 1 Receptor for anti-tumor activity and Thymic Stromal Lymphopoietin cytokine for anti-asthma activity, respectively. Differential scanning calorimetry (DSC) clearly indicates both antibodies in their base formulations have a lower temperature protein conformational change near 70 °C (T_m1_) and a higher temperature protein conformational change near 85 °C (T_m2_). Thermal scanning dynamic light scatting (TS-DLS) indicates a significant particle size increase for both antibodies near T_m2_ suggesting a high level of protein aggregation. The nature of these protein conformational changes associated with increasing the formulation temperature and decreasing sucrose concentration were identified by transmission mode FTIR and second derivative FTIR spectroscopy of temperature controlled aqueous solutions of both monoclonal antibodies. The transition from *intra*-molecular β sheets to *inter*-molecular β sheets was clearly captured for both monoclonal antibodies using FTIR spectroscopy. Finally, FTIR Spectroscopy was able to show the impact of a common excipient such as sucrose on the stability of each monoclonal antibody, further demonstrating the usefulness of FTIR spectroscopy for studying protein aggregation and formulation effects.

## Introduction

Protein aggregation is considered a major formulation problem for therapeutic biologics and vaccines. Most biologic drug product formulations aim to minimize aggregation-induced particle formulation which often reduces the effective dose and could induce an immune response [1]. Many vaccine drug product formulations contain high molecular weight (MW) components and even particulates. However, even for vaccine drug product formulations, excess protein mediated aggregation could severely reduce potency by essentially eliminating antigenic sites for the drug product. Protein aggregation is a complex process often involving a non-Arrhenius process with many pathways which is relatively unique for any specific protein [2]. Protein aggregation is typically quantified by measuring an increase in protein particle diameter in response to stresses such as temperature, time, agitation, freezing, etc. Biologics and vaccine formulation studies often quantify increasing protein particle diameters using techniques such as static light scattering (SLS), dynamic light scattering (DLS), size exclusion chromatograph (HSEC), and microflow imaging (MFI) [3–5]. These and other particle sizing technologies can clearly identify protein aggregation and are very useful screening tools for formulation excipients such as surfactants that inhibit protein aggregation [6–8]. Unfortunately, most particle sizing technology does not provide insight into the specific molecular interactions involved in protein aggregation. However, some bioanalytical techniques such as tryptophan/tyrosine intrinsic protein fluorescence (IPF) and differential scanning fluorimetry (DSF) using extrinsic fluorescence probes can detect general molecular events associated with protein aggregation such as protein unfolding [6, 7].

Anti-IGF1R and anti-TSLP are IgG monoclonal antibodies (mAbs) directed against human Insulin-like Growth Factor 1 Receptor for anti-tumor activity and Thymic Stromal Lymphopoietin cytokine for anti-asthma activity, respectively. The protein unfolding temperatures (T_m_s) of these monoclonal IgG antibodies as determined by differential scanning calorimetry (DSC) were found to be associated with a significant increase in protein particle size by temperature scanning DLS thereby demonstrating protein aggregation. The temperature-dependent changes in the Amide I spectra region of anti-IGF1R and anti-TSLP were examined by both transmission mode FTIR Spectroscopy and 2nd derivative FTIR spectroscopy to identify potential changes in secondary structure associated with protein aggregation. The precise IR adsorption peaks in the Amide I region primarily correspond to the peptide bond C=O stretch vibrations varying with hydrogen bonding in particular protein secondary structures [8]. X-ray diffraction structural analysis shows that the secondary structure of anti-IGF1R and anti-TSLP are 86% and 87% *intra*-molecular β-sheet similar to all IgG antibodies [9–11]. The large amide I FTIR second derivative peak at 1640 cm^−1^ for both antibodies at 25 °C is consistent with a high level of *intra*-molecular β-sheet hydrogen bonding within these IgG antibodies.

Raising either formulation temperature above the IgG T_m2_ results in the large 1640 cm^−1^ Amide I FTIR second derivative peak shifting to 1625 cm^−1^ which has been shown to correspond with the formation of *inter*-molecular β-sheet protein secondary structures [8, 10–13].This suggest that the extensive IgG antibody aggregation above T_m2_ is promoted by the formation of *inter*-molecular β-sheet hydrogen bonding between IgG molecules rather than the *intra*-molecular β-sheet hydrogen of the non-aggregated monoclonal antibodies. Finally, the concentration of sucrose in the monoclonal antibody formulations influenced the magnitude and temperature dependence of the Amide I secondary structure changes, particularly for anti-TSLP. For all of the FTIR spectroscopy studies performed in this paper, computer-based spectral analysis of the temperature dependence of FTIR spectra by QC Compare was utilized and was shown to be consistent with the temperature dependent shifts in the Amide I second derivative adsorption peaks [14].

## Materials and Methods

### Antibodies and Chemicals

The monoclonal antibodies anti-IGF1R and anti-TLSP working stock formulations were provided by Biophysical & Biochemical Characterization, Sterile Formulation Sciences (Merck & Co., Inc., Kenilworth NJ, USA). The working stock formulation of anti-IGF1R was 20 mg/mL protein, 7.0% (w/v) sucrose, 20 mM acetate buffer at pH 5.5. The working stock formulation of anti-TSLP was 40 mg/mL protein, 7.0% (w/v) sucrose, 0.02% (v/v) polysorbate-80, 10 mM histidine buffer at pH 5.5. All other reagents used in this study were BioUltra grade from Sigma Life Sciences (St. Louis, MO).

### Dialysis

Dialysis was performed to change the sucrose concentration for the mAbs studied in this paper. 200 mL of the desired sucrose concentration was placed in a 250 mL beaker with a stir bar. Slide-A-Lyzer Dialysis Mini-tubes (ThermoFisher Scientific Inc., Waltham, MA) with cutoffs anywhere from 3.5 kDa to 10 kDa were utilized. 100 µL of the original mAb solution was placed in the tube and placed in a float until the liquid level in the tube was at the same height as the liquid in the beaker. Beakers were placed on a stir plate in a 4 °C fridge for 12–16 h.

### Dynamic Light Scattering (DLS)

Dynamic light scatting (DLS) was used to track the particle sizes of the protein aggregates as the temperature was increased. Samples were diluted to 4 mg/mL and then loaded into 0.22 µm centrifuge filters and centrifuged at 12,000×*g* for 3 min to get rid of dust particles. 40 µL of sample was added to a 384 well microtiter plate and then centrifuged at 300×*g* for 30 s to get rid of air bubbles. 7 µL of paraffin oil was added to the top of each sample to prevent evaporation; plate was centrifuged again at 300×*g* for 30 s. Samples were loaded into the DynaPro DLS Plate Reader (Wyatt Technology) and heated from 25 to 80 °C (due to machine/programming limitations, samples could not be heated past 80 °C). Instrument was programmed to take 3 DLS measurements of each sample every 0.5 °C.

### Differential Scanning Calorimetry (DSC)

Differential Scanning Calorimetry (DSC) was performed to determine which protein unfolding temperature (T_m_) resulted in more structural changes and unfolding. Measurements for anti-TSLP and anti-IGF1R mAbs were made using a MicroCal VP-Capillary DSC, from Malvern Panalytical (Almelo, The Netherlands). DSC profiles were monitored relative to the background buffer without the mAbs. Samples were diluted to 1 mg/ml and monitored over a temperature range of 25–95 °C at a scan rate of 60 °C/h and resulting data were background corrected.

### FTIR Spectroscopy

The Prota-3S (BioTools, Inc.) FTIR Spectrometer was utilized in this study to collect and analyze FTIR Spectra. For the FTIR studies, anti-IGF1R solutions had a protein concentration of 20 mg/mL while anti-TSLP was at 40 mg/mL. First, the BioCell (composed of CaF_2_ circular plates) with no liquid sample was loaded into the Prota-3S and a “background spectra” was collected. Next, the matching buffer (no protein) was added to the BioCell and a “buffer spectra” was collected. Then, a spectrum of the buffer at low purge (5 SCFH or less) was collected. By setting the buffer spectra at high purge as the background spectra for the buffer spectra at low purge, one acquires a “vapor spectra”. Finally, 25 µL of the protein sample was loaded onto the BioCell and the “protein spectra” was collected. To get the final FTIR Absorbance spectra, the Prota-3S software utilizes the vapor and buffer spectra and subtracts them. Buffer and vapor subtractions are done using a linear regression algorithm, based off the work of Dousseau et al. [15]. The temperatures for all of the spectra collected and described above were matched to the desired temperature of the protein spectra to within 0.3 °C. For all FTIR spectra collected on the Prota-3S the TempCon-2X from BioTools, Inc. was used for temperature control. The TempCon-2X allows for consistent temperature control with a range of − 5 °C to 95 °C.

### Analysis of FTIR Spectra and QC Compare

Spectral analysis of the buffer and water vapor subtracted anti-IGF1R and anti-TSLP protein only spectra recorded with the Prota-3S FTIR instrument was done using the Omnic 8.3 FTIR software package from ThermoFisher Scientific Inc. (Waltham, MA). The 4 cm^−1^ resolution FTIR anti-IGF1R and anti-TSLP spectra from the Prota-3S instrument were analyzed from 1750 to 1450 cm^−1^ by the Omnic FTIR software. This limited the final spectra analysis to just the Amide I and Amide II region of the spectra. A Savitsky–Golay 7 point, 3rd order polynomial, 2nd derivative algorithm was used to generate the 2nd derivative spectra of the monoclonal antibodies. In order to make the magnitude of the 2nd derivative peaks corresponding to IR adsorption peaks positive, each 2nd derivative spectrum was multiplied by − 1. The 2nd derivative peaks in protein FTIR Amide I spectra were used to identify protein secondary structure in the samples. The algorithm QC Compare in the TQ Analyst 8 software package (ThermoFisher Scientific Inc., Waltham, MA) was used to make quantitative comparisons between different FTIR and 2nd derivative FTIR spectra [14]. This software compares the 2nd derivative FTIR spectra of any test sample to the 2nd derivative FTIR spectra of a pre-defined standard without regard to the source of spectral change. The QC Compare quantitative test sample score equals 100 if the 2nd derivative FTIR spectrum of the test sample is a perfect match to the standard spectra even if the sample concentrations are different. The quantitative QC Compare test sample score declines to 0 as the spectral differences between the test and standard spectra increase. The QC Compare algorithm can be used to quantitatively compare stressed sample spectra to standard control samples [16].

## Results

### ***Determining Protein Unfolding Temperatures (T***_***m***_***s) for Anti-IGF1R and Anti-TSLP mAbs***

The protein unfolding temperatures (T_m_s) for the two monoclonal antibodies (mAbs) studied in this paper were first identified through two methods: differential scanning calorimetry (DSC) and dynamic light scattering (DLS). Both of these methods can determine the different T_m_s in addition to the relative magnitudes of unfolding/aggregation occurring at the different T_m_s. However, neither of these methods gives insight into the exact nature of the aggregation such as the kind of conformational change that occurs, etc. For the DSC and DLS experiments, the anti-IGF1R and anti-TSLP mAbs were tested at their standard formulation (pH 5.5, 7% sucrose).

**Fig. 1:**
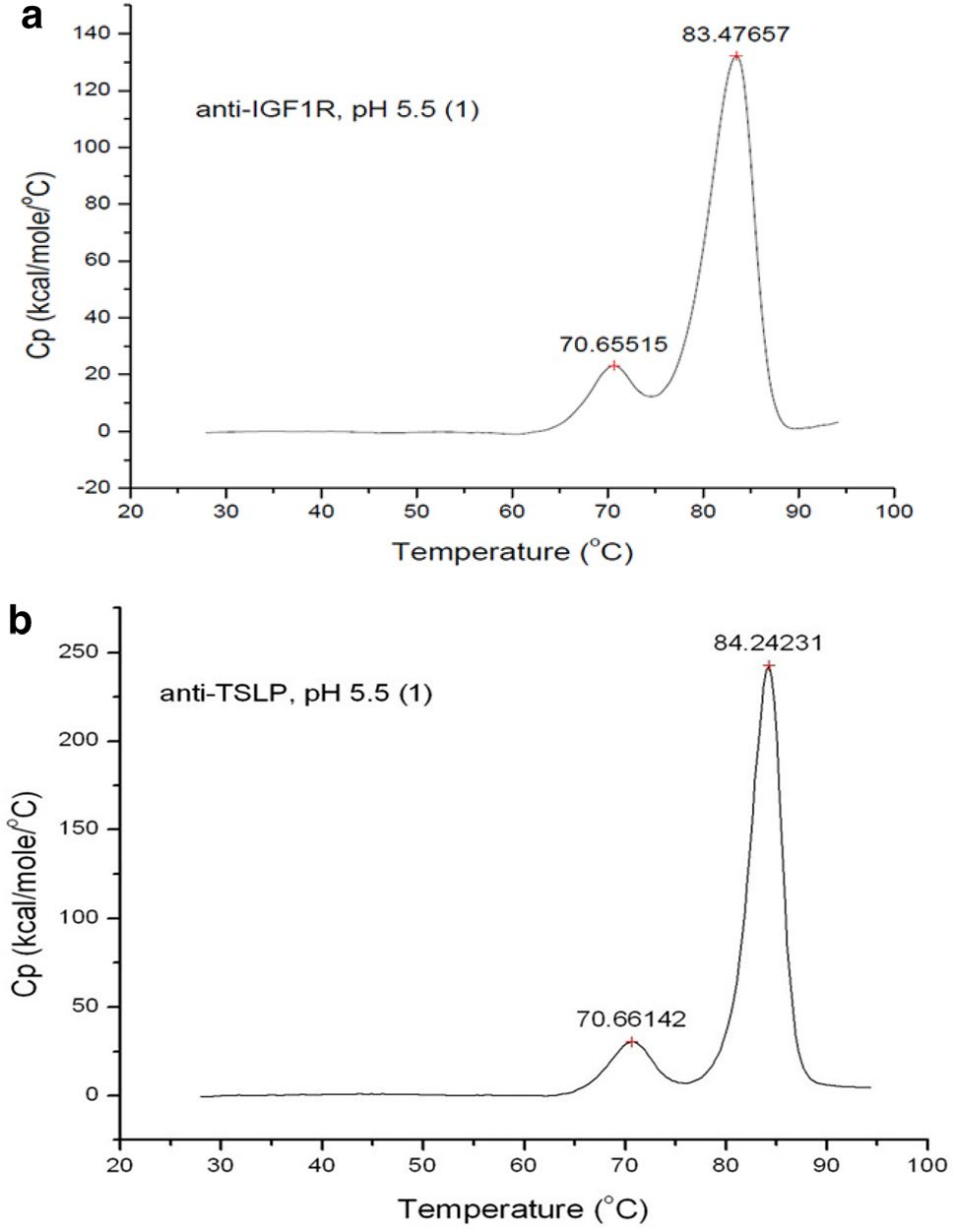
Differential Scanning Calorimetry (DSC) results for anti-IGF1R (**a**) and anti-TSLP (**b**). Both mAbs were studied using DSC to determine the T_m_s in addition to relative magnitudes (degree of aggregation/conformational change) of the T_m_s. Both mAbs were tested in their standard formulation (pH 5.5, 7% sucrose) and tested against their background buffer to derive the DSC curves. See methods section for more details

Differential scanning calorimetry (DSC) is commonly used to detect phase transitions in a variety of materials as well as measuring the protein unfolding temperature(s) (T_m_s) at which proteins denature or undergo conformational changes [17, 18]. By measuring the amount of heat required to increase the temperature of a sample against a background (reference) sample, DSC curves can be derived with the peaks indicating T_m_s when analyzing protein solutions. Fig. 1a, b show the DSC results for anti-IGF1R and anti-TSLP, respectively. The DSC results demonstrate that each mAb has two T_m_s, with each mAb having similar T_m_s: the first occurring at approximately 70 °C and the second occurring at approximately 84 °C. Likewise, for both mAbs, the second T_m_ results in a much more significant conformational change based on the higher amount of heat required to raise the temperature of the sample for the second T_m_ in comparison to the first T_m_ (approximately a fivefold–sevenfold relative difference in heat capacities). This observation is consistent with what is seen for most IgG mAbs as the first T_m_ corresponds to the constant heavy region of the mAb while the second T_m_ corresponds to the variable domains of the mAb. However, the relative magnitudes of the unfolding and the DSC peaks can vary depending on the IgG mAb being studied [18].

**Fig. 2:**
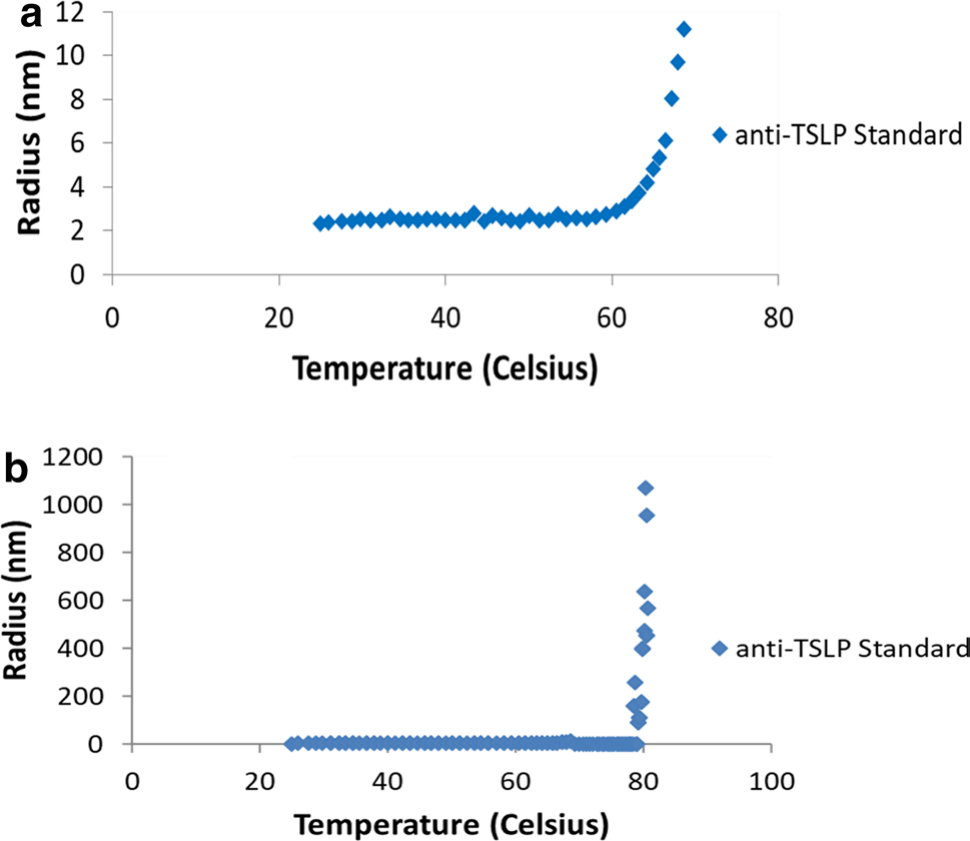
Dynamic Light Scattering (DLS) Results for anti-TSLP at T_m1_ (**a**) and T_m2_ (**b**). DLS studies were done on both anti-TSLP and anti-IGF1R to determine the T_m_s as well as the relative magnitudes of the aggregation/conformational changes. The results for anti-TSLP are shown here, similar results were seen for anti-IGF1R, data not shown. Figure 2A shows the first T_m_ while Fig. 2B shows both T_m_s. However, due to the difference in magnitude between the two T_m_s (measured in terms of average particle radius in nm), the increase in particle size for the first T_m_ cannot be seen in Fig. 2B. The results shown are for anti-TSLP at its standard formulation (pH 5.5, 7% sucrose). Temperatures beyond 80 °C were not tested for the DLS experiments due to DLS machine/programming limitations

Dynamic light scattering (DLS) was also used to determine the T_m_s for the two mAbs studied in this paper as well the relative magnitude of unfolding/aggregation occurring at the T_m_s. DLS is a technique used in physics that can also be utilized in protein studies by studying the distribution of particle sizes in a protein solution over time as the solution is heated [19]. Thus, as the mAb formulations are heated, increases in the average particle size radius indicates protein conformational changes resulting in some degree of protein aggregation. Both anti-IGF1R and anti-TSLP were studied using DLS at their standard formulation (pH 5.5, 7% sucrose). Figure 2 shows the results for anti-TSLP (similar results were observed for anti-IGF1R, data not shown). Figure 2a show the first increase in particle radius occurring at approximately 65–70 °C while Fig. 2b shows the second increase in particle radius occurring at approximately 80 °C. As can be seen in the y-axis scaling for Fig. 2a, b, the second particle size increase (final size ~ 800–1200 nm) is much larger than the first particle size increase (final size 6–12 nm). The particle size distribution of mAb aggregates observed above 80 °C (Fig. 2b) must be very heterogeneous. A large amount of particle size heterogeneity would produce the high variability in DLS particle sizes we observed at 80 °C. This indicates that the second T_m_ results in much more aggregation and protein unfolding than the first T_m_. The DSC and DLS results in Figs. 1 and 2 provide similar results for anti-IGF1R and anti-TSLP in terms of the temperature at which the T_m_s and the relative magnitude of the two T_m_s. However, neither analytical technique provides information as to the exact mechanism as to how the mAbs are unfolding or what kind of conformational change they are undergoing.

### Detecting Intermolecular β-Sheet Formation Using FTIR Spectroscopy

Once DSC and DLS experiments were performed to identify the T_m_s for anti-IGF1R and anti-TSLP as well as the relative magnitudes of the T_m_s (which gives information as to the amount of unfolding/aggregation occurring at the T_m_s), Fourier-Transform Infrared (FTIR) Spectroscopy was performed to gain more insight into the exact mechanism(s) behind the conformational changes and aggregation that is occurring at both T_m_s. FTIR Spectroscopy gives much more information with regards to protein conformational changes compared to DSC and DLS but is a lower throughput technique. However, the combination of these methods might ultimately be very useful for studying protein stability and formulation effects.

**Fig. 3:**
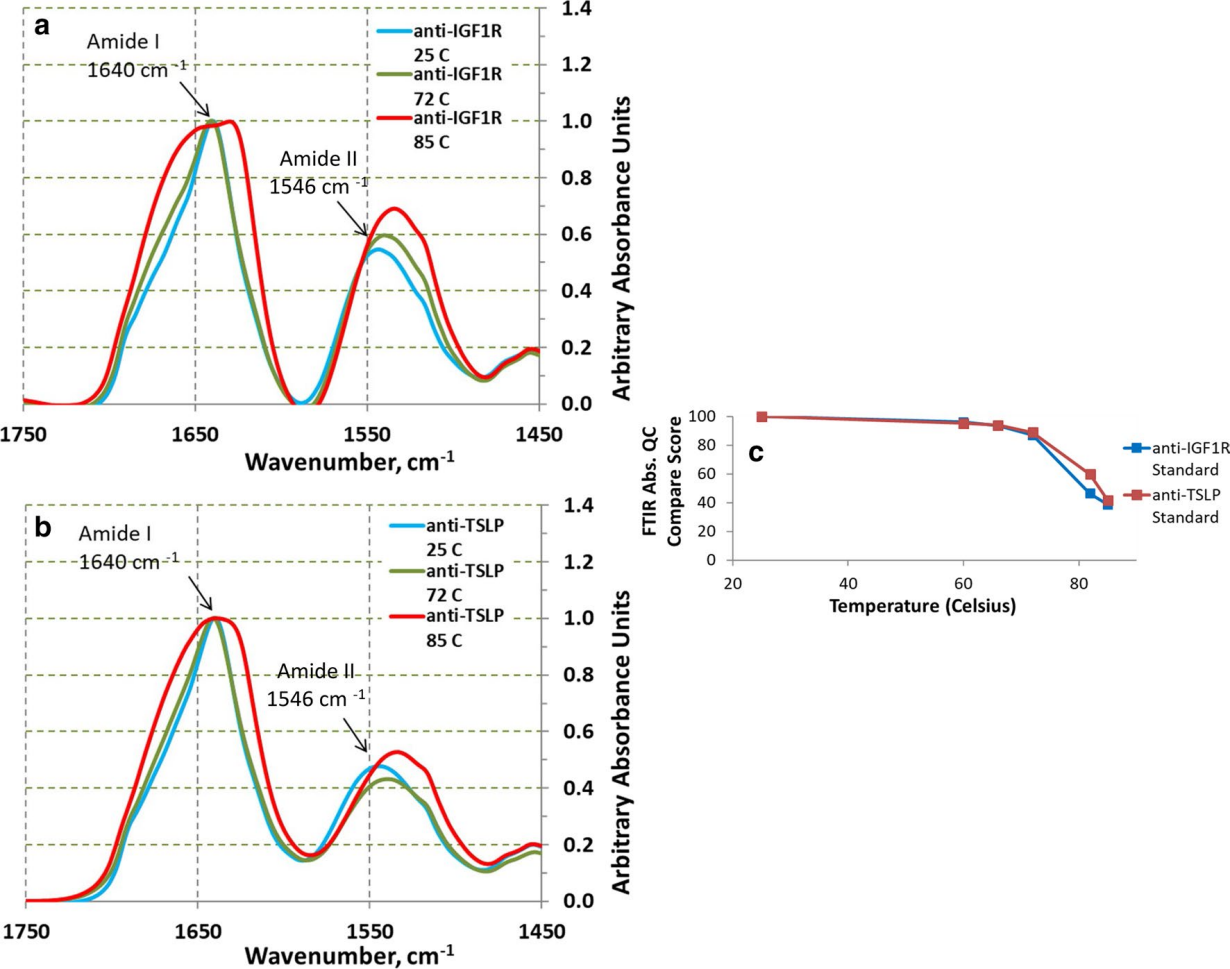
FTIR Absorbance Spectra for anti-IGF1R (**a**) and anti-TSLP (**b**) with QC Compare Scores (**c**). FTIR Absorbance Spectra were collected for anti-IGF1R and anti-TSLP at their standard formulation (pH 5.5, 7% sucrose) and at several temperatures spanning the two T_m_s. See Methods on how the absorbance spectra were collected. **c** shows the QC Comparison Scores for anti-TSLP and anti-IGF1R at the temperatures tested. The spectra at 25 °C for each mAb were used as the “standard” which all spectra were compared to. See Methods for more details about determining QC Compare scores using the TQ Analyst software

First, FTIR absorption spectra were collected for both anti-IGF1R and anti-TSLP at various temperatures (see Methods for more details on how the spectra was collected and analyzed). Several temperatures around the identified T_m_s were selected to analyze anti-IGF1R and anti-TSLP at their standard formulation (pH 5.5, 7% sucrose). Figure 3 shows the results of these experiments at selected temperatures. Figures 3a, b show the FTIR absorbance spectra for anti-IGF1R and anti-TSLP, respectively, at 25 °C, 72 °C, and 82 °C. The FTIR spectrometer software arbitrarily set the maximum absorbance peak value of each spectrum to 1.0 to make qualitative comparisons easier. As can be seen in the spectra, very little changes occur during the first T_m_ (~ 70 °C) but significant structural changes occur after the second T_m_ (~ 84 °C). Heating both anti-IGF1R and anti-TSLP past the second T_m_s results in changes to both the Amide I (1640 cm^−1^) and Amide II (1546 cm^−1^) peaks. The second T_m_ resulting in more significant conformational changes compared to the first T_m_ is consistent with what was observed in the DSC and DLS studies.

Additionally, a simpler way to visualize and quantify the differences in the spectra as the samples are heated is through QC Comparison scores. QC Compare takes the spectrum at each temperature studied and quantitatively compares them to a given standard spectrum. In these studies, the standard protein formulation collected at 25 °C is defined as the “standard” spectrum (see Methods for more details). Thus, as the spectra changes as the sample is heated, the QC compare score will decrease. Figure 3c gives the QC Comparison scores for anti-IGF1R and anti-TSLP as they are heated past both T_m_s. The results in Fig. 3c match what is seen in the absorbance spectra in that more significant changes occur at the second T_m_ than the first T_m_ and that anti-IGF1R and anti-TSLP behave similarly over the temperature range studied. Therefore, the QC Comparison tool is useful for summarizing differences seen in FTIR spectra and quantifying the magnitude of the changes.

**Fig. 4:**
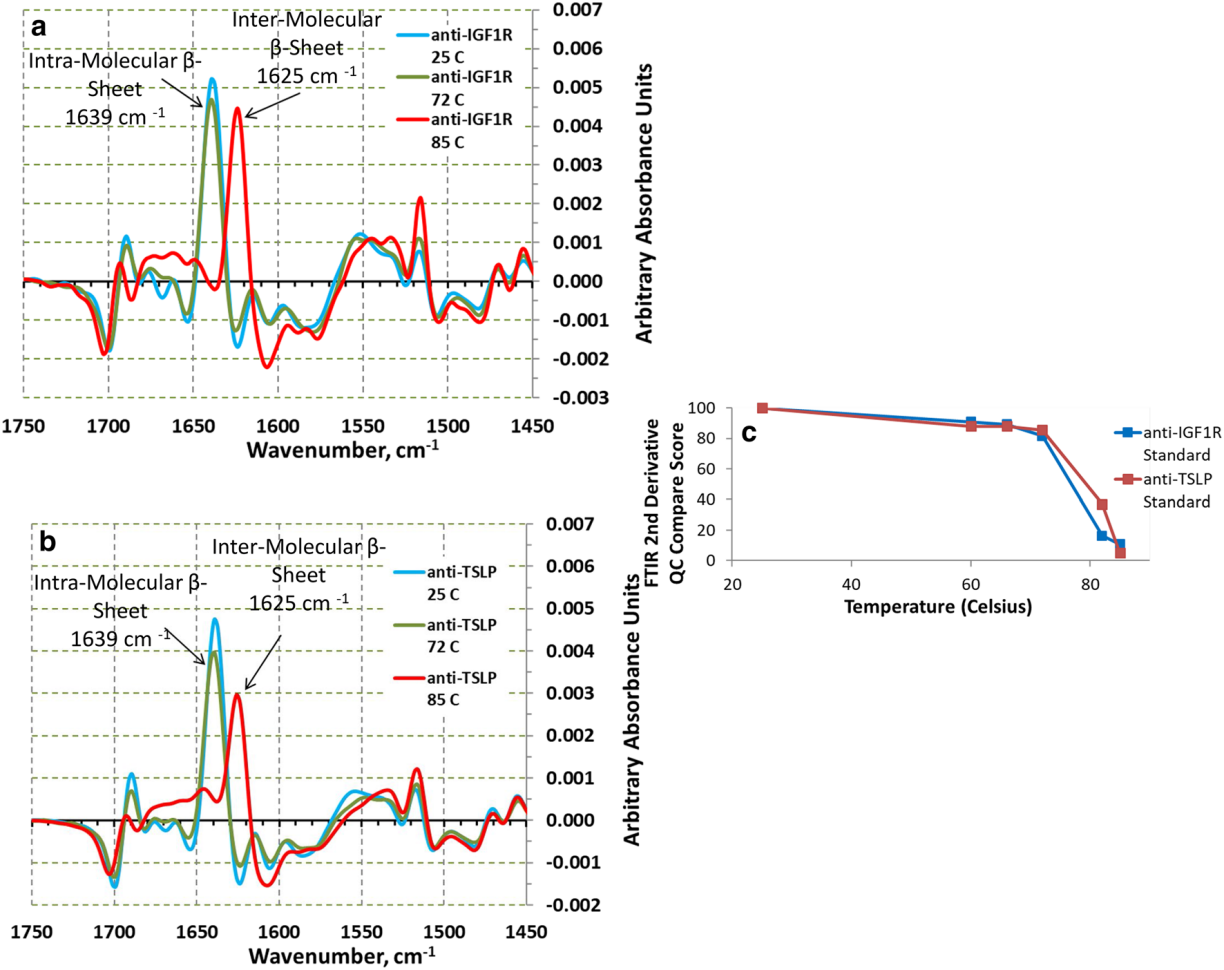
FTIR 2nd Derivative Spectra for anti-IGF1R (**a**) and anti-TSLP (**b**) with QC Compare Scores (**c**). FTIR 2nd Derivative Spectra were collected for anti-IGF1R and anti-TSLP at their standard formulation (pH 5.5, 7% sucrose) and at several temperatures spanning the two T_m_s. 2nd derivative spectra were calculated by using the absorbance spectra seen in Fig. 3. See Methods for more details on how the 2nd derivative spectra were collected. Figure 3C shows the QC Comparison Scores for anti-TSLP and anti-IGF1R at the temperatures tested. The spectra at 25 °C for each mAb were used as the “standard” which all spectra were compared to. See Methods for more details about determining QC Compare scores using the TQ Analyst software

After confirming that the FTIR absorbance spectra for anti-IGF1R and anti-TSLP across different temperatures spanning the two T_m_s resulted in similar conclusions as the DSC and DLS experiments, 2nd derivative FTIR spectra were analyzed. 2nd derivative FTIR spectra provide more information with regards to the protein structure and different elements present in the protein. Peaks at particular wavenumber positions correspond to certain protein structures. Devi et al. [8] among others have compiled a list of wavenumber frequencies corresponding to common mAb secondary structures such as α-helices, β-sheets, random coils, turns, etc. The 2nd derivative spectra are derived by taking the FTIR Absorbance spectra and using the OMNIC software package to find the 2nd derivative peaks as seen in Fig. 4 (see Methods for more details about how the 2nd derivative spectra were collected).

Figure 4 represents the 2nd derivative spectra of the FTIR Absorbance Spectra collected in Fig. 3 across different temperatures spanning the two T_m_s. Figure 4a shows the 2nd derivative spectra for anti-IGF1R at 25 °C, 72 °C, and 82 °C while Fig. 4b shows the 2nd derivative spectra for anti-TSLP at the same temperatures. Like the absorbance spectra, QC Compare scores were also collected for the 2nd derivative spectra to give a more quantitative snapshot of the changes as the samples were heated. Figure 4c contains the QC compare results for the 2nd derivative spectra and confirms many of the same details that the FTIR Absorbance QC Compare scores demonstrated. Most importantly, the second T_m_ results in more conformational changes than the first T_m_. Furthermore, the QC Compare Scores for the 2nd derivative spectra only further magnify the changes that are occurring between the first T_m_ and second T_m_ based on the lower QC Compare scores at higher temperatures.

A closer examination of the 2nd derivative spectra in Fig. 4a, b reveal many intriguing details about the conformational changes occurring as the anti-IGF1R and anti-TSLP samples are heated past the first and second T_m_s. As confirmed by DSC, DLS, and the FTIR absorbance spectra, the first T_m_ results in very little to no conformational changes compared to the formulation at 25 °C. The major peaks in the 2nd derivative spectra remain unchanged in position as the samples are heated up to 72 °C. But as the anti-IGF1R and anti-TSLP samples are heated past the second T_m_ (~ 84 °C), one of the peaks in the Amide I region shifts dramatically. As demonstrated by Devi et al. [8], the peak at 1639 cm^−1^ seen in the 2nd derivative spectra for anti-IGF1R and anti-TSLP up to the second T_m_ signifies *intra*-molecular β-sheet folding present in the protein formulations. But as anti-IGF1R and anti-TSLP are heated past their 2nd T_m_, the peak at 1639 cm^−1^ shifts to 1625 cm^−1^ which indicates *inter*-molecular β-sheet formation in the protein formulations.

This conformational change is consistent with the significant aggregation observed in the DLS experiments. *Intra*-molecular β-sheets are β-sheets formed within individual mAbs or proteins, but *inter*-molecular β-sheets are formed amongst separate mAbs or proteins resulting in protein aggregation or clustering [10, 12]. This observation demonstrates the usefulness of utilizing FTIR spectroscopy and 2nd derivative FTIR spectra to study protein stability and protein aggregation. While DSC and DLS experiments provided information as to what temperatures conformational changes or aggregation was occurring in addition to the relative magnitudes, neither method provided insight into the exact conformational changes that were occurring. Determining and understanding what kinds of conformational changes are occurring at different T_m_s for mAbs and other proteins could be very important for studying protein stability and screening for stable protein formulations (see "Discussion/Conclusion" for a more details conversation around this proposal).

### Effect of Sucrose Concentration in Stabilizing Anti-IGF1R and Anti-TSLP

After demonstrating that FTIR spectroscopy could be used to determine the specific conformational changes occurring at different protein unfolding temperatures for anti-IGF1R and anti-TSLP mAbs, we employed FTIR spectroscopy to study formulation effects of sucrose concentration on these two mAbs. The amount of sucrose in the anti-IGF1R and anti-TSLP formulations was reduced through dialysis. We selected three different levels of sucrose concentrations (No sucrose, 1.5% sucrose, and 7% sucrose) to study with FTIR spectroscopy to see how sucrose impacts the conformational changes we saw with the standard formulations of anti-IGF1R and anti-TSLP.

**Fig. 5:**
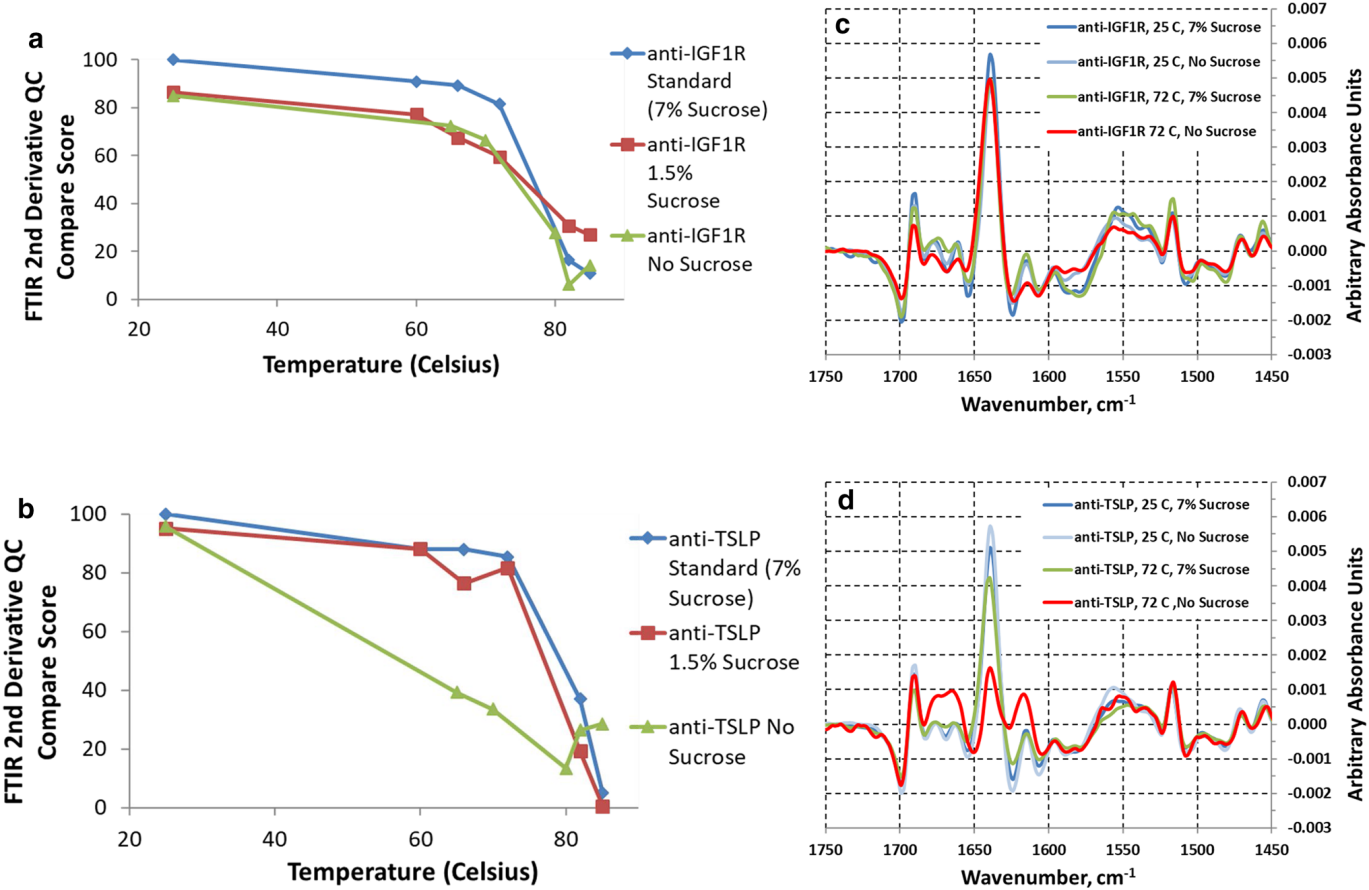
FTIR 2nd Derivative QC Compare Scores for anti-IGF1R (**a**) and anti-TSLP (**b**) with different sucrose concentrations, 2nd Derivative Spectra for anti-IGF1R (**c**) and anti-TSLP (**d**) with 7% sucrose and no sucrose. Anti-TSLP and anti-IGF1R formulations with different sucrose concentrations were heated and FTIR Absorbance spectra were calculated at the same temperatures studied in Figs. 3 and 4. 2nd Derivative spectra were collected from the absorbance spectra as described in Methods and QC Compare scores were derived. The mAbs (anti-IGF1R and anti-TSLP) with a standard sucrose concentration of 7% at 25 °C were used as the “standards” for which the rest of the spectra (at different temperatures and sucrose concentrations) were compared to. Figure 5a, b show the QC Compare results while Fig. 5c, d show the 2nd derivative spectra for anti-IGF1R and anti-TSLP, respectively, with 7% sucrose and no sucrose at room temperature (25 °C) and at 72 °C (just past the usual first T_m_ for these mAbs)

As done previously, FTIR Absorbance spectra were collected for the different formulations of anti-IGF1R and anti-TSLP at multiple temperatures spanning the two T_m_s. Once the FTIR absorbance spectra were collected, 2nd derivative spectra and QC Comparison scores were collected. Fig. 5a, b show the QC comparison results for the anti-IGF1R and anti-TSLP 2nd derivative spectra, respectively. The QC comparison scores give a good quantitative overview of the changes occurring as the different formulations are heated past their two T_m_s. Note that the standard formulations (7% sucrose) for anti-IGF1R and anti-TSLP at 25 °C were used as the “standards” which all of the other spectra (different sucrose concentrations, different temperatures) were compared to. This explains why the QC Compare scores at 25 °C are different for the different amounts of sucrose: the amount of sucrose in the formulation has a slight initial impact on the 2nd derivative spectra for each of the formulations and the QC Compare Scores capture these differences. Overall, for both anti-IGF1R and anti-TSLP, similar results as before are seen for the 7% (standard) and 1.5% sucrose formulations, i.e. significant conformational changes occur after the second T_m_.

The formulations with no sucrose demonstrate how sucrose stabilizes anti-IGF1R and anti-TSLP, particularly anti-TSLP. The QC Comparison scores for the anti-TSLP formulation with no sucrose are dramatically different than the QC Compare scores for the 1.5% and 7% sucrose anti-TSLP formulations (Fig. 5b). It can be seen that even before the usual first T_m_ position (~ 70 °C), significant conformational changes are occurring as demonstrated by the decrease in QC Comparison scores. The 2nd derivative spectra for the standard formulation of anti-TSLP (7% sucrose) and anti-TSLP with no sucrose at 25 °C and 72 °C are shown in Fig. 5d. The spectra reveal that at 72 °C, the anti-TSLP formulation with no sucrose is already beginning to form inter-molecular β-sheets as evidenced by the formation of a peak at 1625 cm^−1^. For the other concentrations of sucrose tested, the formation of inter-molecular β-sheets is not seen until after or around the second T_m_ (~ 80–85 °C, data not shown). Thus, sucrose appears to have a major effect in stabilizing anti-TSLP and preventing the formation of inter-molecular β-sheets at T_m1_.

A similar effect was not observed with anti-IGF1R. The QC Compare scores (Fig. 5a) are similar for the three different sucrose formulations suggesting that sucrose plays less of a role in preventing inter-molecular β-sheets at T_m1_. Figure 5c shows the 2nd derivative spectra for the standard formulation of anti-IGF1R (7% sucrose) and anti-IGF1R with no sucrose at 25 °C and 72 °C. These figures backs up the results captured in the QC Compare Scores as little changes are seen in the spectra with no sucrose compared to the standard formulation (7% sucrose) as the samples are heated past the first T_m_. Overall, this set of experiments demonstrates the usefulness of using FTIR Spectroscopy to study the conformational changes occurring at different T_m_s as the formulation is changed.

## Discussion

Beginning with the ground-breaking deconvolved FTIR protein spectra analysis of Byler and Susi, numerous FTIR techniques have been applied to understanding the secondary structure of proteins [20, 21]. Many FTIR studies of proteins have associated 2nd derivative FTIR spectra peaks at 1640 and 1625 cm-1 with intra-molecular and inter-molecular β-sheets respectively [8, 11–13]. The assignment of a particular secondary structure to a specific frequency peaks has been made in reference to known three-dimensional proteins structures, synthetic peptides, and theoretical calculations. Amide I intra-molecular and inter-molecular β-sheets are considered to be anti-parallel and parallel β-sheets, i.e. extended strand, protein structures, respectively [11, 22]. Formation of the 1620 to 1630 cm^−1^ 2nd derivative FTIR peak has been associated with protein aggregation due to strong organic solvents and lyophilization [10, 12, 13]. Temperature induced 1620 to 1630 cm^−1^ peaks were also observed above the apparent T_m_s of several proteins including IgG immunoglobulin consistent with our results [8, 11]. Recent attenuated total reflectance FTIR (ATR-FTIR) and FTIR microscopy studies have provided strong evidence for β-sheet mediated protein aggregation [23–25]. Our DLS and FTIR results with these two monoclonal antibodies further demonstrate the importance of inter-molecular β-sheet formation in temperature-dependent protein aggregation.

Both anti-IGF1R and anti-TSLP exhibit two thermally induced protein unfolding events during DSC scans similar to many monoclonal IgG as shown in Fig. 1 [18]. It seems apparent that the majority of the IgG intra-molecular β-sheets must unfold at the higher temperature larger unfolding transition, T_m2_, for both mAbs as indicated in Fig. 1. Once the *intra*-molecular β-sheets hydrogen bonding is disrupted by heating above a certain temperature, new *inter*-molecular β-sheets hydrogen bonding can form. Both anti-IGF1R and anti-TSLP form inter-molecular associations and appear to aggregate above their high T_m2_ through inter-molecular β-sheet contacts as indicated in Figs. 3 and 4. The DLS measurements shown in Fig. 2 show that these inter-molecular contacts allow for the formation of very large protein aggregates; aggregating to particle sizes up to hundreds of nm in diameter.

Although similar, the nature of the thermal-induced secondary structure changes in the two mAbs identified by FTIR are not identical. In particular, 7% (w/v) sucrose is apparently required to maintain significant intra-molecular β-sheet structure of anti-TSLP through the T_m1_ transition, but not for anti-IGF1R as it goes through T_m1_ (see Fig. 5). Both mAbs lose intra-molecular β-sheet secondary structure above T_m2_ with or without 7% (w/v) sucrose. This small example demonstrates how information-rich temperature-scanning 2nd derivative FTIR spectroscopy can identify important protein structural changes that are not apparent with DSC or DSF. It appears that even sucrose concentrations below 7% (w/v) can stabilize anti-TSLP through the T_m1_ transition (see Fig. 5b). Sucrose is probably not the only excipient or even the best excipient that can stabilize anti-TSLP through T_m1_ by inhibiting inter-molecular β-sheet formation. Other potential intra-molecular β-sheet stabilizing formulation excipients should be now screened in more easily automated methods for increasing the T_m1_ and T_m2_ temperature in future studies. We have already completed an automated high-throughput DSF screen of anti-IGFR1 with 23 excipients at 4 different concentrations, each in a 96 well plate. Xylitol, sorbitol, trehalose all increased T_m2_, but sucrose was the most effective (unpublished results). Thus, this specific example shines light on how FTIR spectroscopy can enhance formulation development of monoclonal antibodies. This formulation development concept is further discussed below.

Taking the 2nd derivative of FTIR protein Amide I spectra is a common and well-established procedure to identify the position of particular IR adsorption peaks in the complicated and overlapping collection of IR adsorption bands. A large amount of research by many individuals has associated particular protein structures with specific IR adsorption peak identified by 2nd derivative FTIR [8, 11–13, 15]. As discussed above, we have taken advantage of all this research to qualitatively identify specific thermally induced structural changes in anti-IGF1R and anti-TSLP. A simple quantitative comparison of anti-IGFR and anti-TSLP IR 2nd derivative spectra were done in this study using QC Compare. This software has been shown to be a sensitive and precise method to compare the FTIR 2nd derivative spectra of stressed monoclonal antibodies [16]. Plotting the monoclonal QC Compare scores as a function of temperature using the 25 °C FTIR spectra as a standard quantitatively describes how the FTIR spectra of anti-IGF1R and anti-TSLP change with temperature (see Figs. 3, 4 and 5).

## Conclusion

Temperature controlled transmission mode FTIR spectroscopy of protein biologics and vaccine antigens can provide a wealth of information on protein secondary structure. We have shown in this report that transmission mode IR spectroscopy can provide much detailed information on stress induced protein conformation changes. These changes can also help identify formulation excipients, e.g. sucrose, that prevent or mitigate the stress-induced changes some of which can lead to protein aggregation. Rapid high-throughput formulation screening by the conventional aqueous transmission mode FTIR protein spectroscopy as described in this report spectroscopy can be technically challenging. However, automated IR protein formulation screening using microfluidic modulation technology should allow more rapid IR formulation screening [26]. We propose accelerating and enhancing future formulation development for both biologics and vaccines by combining conventional aqueous transmission mode FTIR with recently developed microfluidic modulation IR spectroscopy. This approach should allow for rapid stability optimization of biologic and vaccine formulation using novel excipients and stress stability conditions.
